# Echocardiographic myocardial strain analysis describes subclinical cardiac dysfunction after craniospinal irradiation in pediatric and young adult patients with central nervous system tumors

**DOI:** 10.1186/s40959-021-00093-z

**Published:** 2021-02-02

**Authors:** Hugo R. Martinez, Ralph Salloum, Erin Wright, Lauren Bueche, Philip R. Khoury, Justin T. Tretter, Thomas D. Ryan

**Affiliations:** 1 Division of Cardiology, Department of Pediatrics, University of Cincinnati College of Medicine, and Heart Institute, Cincinnati Children’s Hospital Medical Center, 3333 Burnet Ave, MLC 2003, Cincinnati, OH 45229 USA; 2 Present address: Division of Pediatric Cardiology, Heart Institute, Le Bonheur Children’s Hospital, University of Tennessee Health and Science Center, Memphis, TN USA; 3 Division of Oncology, Department of Pediatrics, University of Cincinnati College of Medicine, and Cancer and Blood Disorders Institute, Cincinnati Children’s Hospital Medical Center, Cincinnati, OH USA; 4 Present address: Division of Hematology/Oncology/Bone Marrow Transplant Nationwide Children’s Hospital Columbus, Columbus, OH USA; 5 Present address: Division of Hematology and Oncology, Showers Family Center for Childhood Cancer and Blood Disorders, Akron Children’s Hospital, Akron, OH USA

**Keywords:** Craniospinal irradiation, Neuro-oncology, Cardiac, Surveillance

## Abstract

**Background:**

Craniospinal irradiation (CSI) is part of the treatment of central nervous system (CNS) tumors and is associated with cardiovascular disease in adults. Global myocardial strain analysis including longitudinal peak systolic strain (GLS), circumferential peak systolic strain (GCS), and radial peak systolic strain (GRS) can reveal subclinical cardiac dysfunction.

**Methods:**

Retrospective, single-center study in patients managed with CSI vs. age-matched controls. Clinical data and echocardiography, including myocardial strain analysis, were collected at early (< 12 months) and late (≥ 12 months) time points after completion of CSI.

**Results:**

Echocardiograms were available at 20 early and 34 late time points. Patients at the late time point were older (21.7 ± 10.4 vs. 13.3 ± 9.6 years) and further out from CSI (13.1 ± 8.8 vs. 0.2 ± 0.3 years). Standard echocardiographic parameters were normal for both groups. For early, CSI vs. control: GLS was − 16.8 ± 3.6% vs. -21.3 ± 4.0% (*p* = 0.0002), GCS was − 22.5 ± 5.2% vs. -21.3 ± 3.4% (*p* = 0.28), and GRS was 21.8 ± 11.0% vs. 26.9 ± 7.7% (*p* = 0.07). For late, CSI vs. control: GLS was − 16.2 ± 5.4% vs. -21.6 ± 3.7% (*p* < 0.0001), GCS was − 20.9 ± 6.8% vs. -21.9 ± 3.5% (*p* = 0.42), and GRS was 22.5 ± 10.0% vs. 27.3 ± 8.3% (*p* = 0.03). Radiation type (proton vs. photon), and radiation dose (< 30 Gy vs. ≥ 30 Gy) did not impact any parameter, although numbers were small.

**Conclusions:**

Subclinical cardiac systolic dysfunction by GLS is present both early and late after CSI. These results argue for future studies to determine baseline cardiovascular status and the need for early initiation of longitudinal follow-up post CSI.

## Introduction

Each year there are over 1.7 million new cases of cancer in the United States, including almost 16,000 pediatric patients (< 20-years-old). Advances in treatment have led to 5-year survival of > 80% for all pediatric cancer types, which amounts to more than 450,000 total survivors of pediatric cancers [1, 2]. Development of cardiovascular disease is the leading non-cancer cause of morbidity and mortality in this population, and radiation therapy has been reported to increase the risk of cancer related cardiotoxicity through injury of the pericardium, coronary arteries, valves, conduction system, and the myocardium [3]. Few studies have looked exclusively at patients with central nervous system (CNS) cancers and exposure to craniospinal irradiation (CSI), which is known to lead to out-of-field exposure of cardiovascular tissues [4, 5]. Surveillance guidelines for survivors of childhood cancer recommend screening for cardiotoxicity, including echocardiogram every 2–5 years based on risk factors, e.g., total cumulative anthracycline dose and/or radiation exposure [6]. Although echocardiographic ejection fraction (EF) and shortening fraction (SF) have traditionally served to monitor left ventricular systolic function and guide clinical judgement during and after cancer-related therapies, both EF and SF are influenced by ventricular preload and afterload and may not reflect ventricular contractility as much as they represent ventricular remodeling [7]. Additionally, SF and EF are operator dependent and rely on geometric assumptions, with poor reproducibility reported in a large multi-center study of healthy pediatric patients [8]. Assessment of tissue deformation by speckle tracking echocardiography may better assess left ventricular contractility with good reproducibility [9], and although influenced by afterload, has demonstrated the ability to detect subclinical dysfunction after cancer treatment in both pediatric and adult patients [10–12]. No prior studies have assessed temporal changes in left ventricular systolic function in patients with exposure to CSI in the setting of CNS malignancies. We postulated that exposure to CSI would lead to evidence for cardiac injury, either clinical or subclinical, and aimed to evaluate such changes by two-dimensional speckle tracking echocardiography in a retrospective pediatric and young adult cohort with CNS malignancies.

## Methods

This was a retrospective study performed at a single-center, Cincinnati Children’s Hospital Medical Center. Patients diagnosed with CNS malignancy and managed with CSI between the years 1986–2018 were identified by the Neuro-Oncology Program and included in the study if they had at least one echocardiogram post therapy with digitized images available for review by speckle tracking. Patients who received anthracyclines were excluded from the study. Transthoracic echocardiograms were performed on 1 of 3 ultrasound systems used at our institution during the study period: Vivid 7 (General Electric Healthcare, Milwaukee, WI), iE33 (Phillips Medical Systems, Best, The Netherlands), and Sequoia 512 (Acuson, Oceanside, CA). Demographic, treatment (radiation dosing, anthracycline exposure), relevant medical history, relevant lab values, and standard echocardiographic data were collected from the electronic medical record for two time periods: “early” representing an echocardiogram performed at < 12 months from the end of therapy, and “late” representing an echocardiogram performed at ≥ 12 months from the end of therapy. Of note, very few patients had echocardiography performed prior to CSI so this time point was not included. For any missing echocardiographic data (i.e., SF and EF), measurements were made retroactively. These data were compared to well-established population normal values.

Myocardial strain is a relatively angle-independent measure of ventricular function, noninvasively measured by echocardiogram or cardiac MRI [13]. Strain is reported as a percent and can be measured in a global or segmental fashion, in one of three planes: longitudinal (negative value), circumferential (negative value), and radial (positive value). For evaluation of global longitudinal strain (GLS), global circumferential strain (GCS), and global radial strain (GRS) by two-dimensional speckle tracking echocardiography, *post-hoc* analysis was performed at early and late time points. In brief, Digital Imaging and Communications in Medicine (DICOM) data were analyzed using vendor-independent clinical echocardiographic software (Image Arena, TomTec Imaging Systems, Munich, Germany). Since there are no available normative data for two-dimensional strain in pediatric patients specific to this software, an age-matched control group was generated from individuals referred for echocardiogram for a variety of reasons (e.g., family history of cardiomyopathy, murmur) and ultimately found to have no cardiac pathology. Because these individuals had normal echocardiograms, in almost all cases no further laboratory data or work up was pursued and thus there was no evaluation for factors that may affect strain such as hemoglobin. However, it was assumed these patients, as a group, were normal regarding medical history and laboratory data.

Data were examined for completeness, distributions were examined for shape, and outliers were checked for feasibility. Cross-checks were done, including by scatterplots, and contingency tables. Any questionable data points were referred back to the source, and any typographical, or other errors in reporting were corrected. Patient characteristics and demographics were displayed in contingency tables, and as means ± standard deviations for continuous variables. A control group was established for each time point by selecting control echocardiographic studies to match patients as closely as possible by age. Comparisons between controls and patients were done using Fisher’s exact tests for categorical data, and continuous variables were tested using Wilcoxon rank-sums analyses. Boxplots were constructed to display differences between control and patient results. Comparisons between early and late results were done using paired t-tests.

## Results

A total of 51 patients treated with CSI for CNS malignancies were identified from our database for the years 1986–2018, including 67% (34/51) male and 88% (45/51) white race. The most common diagnosis was medulloblastoma (45/51), with the remaining cases including atypical teratoid, ependymoma, and glioma. Most patients received cisplatin (44/51) and cyclophosphamide (39/51). While almost all patients received craniospinal radiation, 1 received only spinal radiation and 1 received no spinal radiation. The latter patient was excluded from further analysis. Photon therapy was used more often than proton, and more than half of the patients received ≥ 30 Gy of spinal radiation (Table 1).

**Table 1:** Patient and disease characteristics at enrollment. *N* = 50 patients studied

	*Male*	*Female*			
**Sex**	34	17			
	*White*	*Black*	*Asian*	*Other*	
**Race**	45	4	1	1	
	*Medulloblastoma*	*Atypical Teratoid*	*Ependymoma*	*Glioma*	*Other*
**Diagnosis**	42	2	1	2	4
	*Brain*	*Spine, cervical*	*Spine, Thoracic*	*Spine, Lumbar*	*Other*
**Cancer Location**^**a**^	51	2	1	4	11
	*Yes*	*No*			
**Chemotherapy Used**	49	2			
	*Cisplatin*	*Cyclophosphamide*	*Carboplatin*	*Lomustine*	*Ifosfamide*
**Chemotherapy Type**	44	39	19	19	1
	*Craniospinal*	*Spinal Only*			
**Radiation Location**	50	1			
	*Photon*	*Proton*	*Unknown*		
**Radiation Type**	36	14	1		
	*> 0 to < 30*	≥*30*	*None*^*b*^	Unknown	
**Radiation Dose to the spine, Gy**	17	29	1	4	

^a^Other locations included: leptomeningeal (5), positive cerebrospinal fluid (4), sacral (2)

^b^Patient removed from analysis

Echocardiograms were available in 20 patients at the early time point and 34 patients at the late time point. There were 13 patients with echocardiograms in both groups, however the time points were not compared to each other and considered standalone time points. The oldest echocardiogram included in analysis was performed in 2007. Patients at the late time point were older than those in the early group (21.7 ± 10.4 vs. 13.3 ± 9.6 years, respectively), and time between end of therapy and echocardiogram was greater (13.1 ± 8.8 vs. 0.2 ± 0.3 years, respectively) (Table 2). Standard echocardiographic parameters assessing left ventricular systolic function were normal for all subjects when compared to age-based published normal values. In the late group, two patients were on the combination of lisinopril and carvedilol for a history of mild left ventricular dysfunction, and one was on atenolol for sinus tachycardia and diastolic dysfunction on echocardiogram. Afterload, represented by systolic blood pressure, was normal for both groups. In the early group one patient was on atenolol for hypertension, and in the late group two patients had systolic blood pressure greater than 95%ile but they were not on medication. Parameters known to affect ventricular function such as hemoglobin (anemia) and creatinine (renal dysfunction) were within normal limits for both groups. One patient in the late group was on metformin for diabetes management (Table 2).

**Table 2:** Demographic, laboratory, and standard echocardiographic measures at early and late timepoints after craniospinal radiation (see text for definition of terms)

	*Early*	*Late*
**N**	20^a^	34^b^
**Age (years)**	13.3 ± 9.6	21.7 ± 10.4
**Time since therapy completion (years)**	0.2 ± 0.3	13.1 ± 8.8
**SBP (mmHg)**	105.4 ± 10.3	115.2 ± 16.1
**LVEDD (mm)**	4.0 ± 0.6	3.9 ± 0.6
**LVFS (%)**	32.6 ± 5.4	34.4 ± 3.8
**LVEF (%)**	62.3 ± 6.5	60.9 ± 4.5
**Presence of diabetes mellitus**^**c**^	0 (0%)	1 (3%)
**Presence of hypertension**^**d**^	1 (5%)	2 (6%)
**Patient on cardiac medication(s)**^**e**^	1 (5%)	3 (9%)
**Hemoglobin (g/dL)**^**f**^	11.3 ± 1.5	13.5 ± 2.3
**Creatinine (mg/dL)**^**f**^	0.5 ± 0.1	0.7 ± 0.3

^a^N available for analysis: LVEDD = 19; LVEF = 15; all other = 20

^b^N available for analysis: SBP and LVSF = 33; LVEDD and LVEF = 30; hemoglobin = 29; creatinine = 27; all other = 34

^c^Patients with a diagnosis of diabetes mellitus documented in the chart or on medication to control diabetes at the time of the echocardiogram

^d^Patients with systolic blood pressure > 95%ile for age or on antihypertensive medication at the time of the echocardiogram

^e^Patients on cardiac medication(s) at time of echocardiogram, including angiotensin converting enzyme inhibitor, angiotensin receptor blocker, beta blocker, calcium channel blocker, or mineralocorticoid receptor antagonist

^f^Laboratory data collected from the electronic medical record for the value closest in time to the echocardiogram but no greater than ± 8 weeks

*LV* Left ventricular, *LVEDD* Left ventricular diastolic dimension, *LVEF* Left ventricular ejection fraction, *LVFS* Left ventricular fractional shortening, *SBP* Systolic blood pressure

When considering myocardial deformation measured by myocardial strain analysis, at early, CSI vs. control: GLS was − 16.8 ± 3.6% vs. -21.3 ± 4.0% (*p* = 0.0002), GCS was − 22.5 ± 5.2% vs. -21.3 ± 3.4% (*p* = 0.28), and GRS was 21.8 ± 11.0% vs. 26.9 ± 7.7% (*p* = 0.07). At late, CSI vs. control: GLS was − 16.2 ± 5.4% vs. -21.6 ± 3.7% (*p* < 0.0001), GCS was − 20.9 ± 6.8% vs. -21.9 ± 3.5% (*p* = 0.42), and GRS was 22.5 ± 10.0% vs. 27.3 ± 8.3% (*p* = 0.03) (Table 3). Because a cut-off of 30 Gy radiation to the heart is a known risk factor for development of cardiotoxicity [6], we assessed the differential effect of low-dose CSI (< 30 Gy) and high-dose CSI (≥ 30 Gy) on strain parameters in the study. In addition, with decreased off-target effects reported for proton vs. photon sources of radiation [4] we assessed whether there was a difference in strain values between patients treated with these two modalities. There were no statistically significant differences between either of these treatment conditions at either time point for any of the strain values (Table 3).

**Table 3:** Global longitudinal strain (GCS), global circumferential strain (GCS), and global radial strain (GRS) in patients vs. age- and sex-matched controls at early and late (see text for definition), photon vs. proton radiation type, and spinal radiation exposure of < 30 Gy vs. ≥ 30 Gy

	*Early*	*Control*	*p-value*	*Late*	*Control*	*p-value*
**N**	19	36	–	30	48	–
**Male**	10 (52%)	23 (64%)	0.56	21 (70%)	31 (65%)	0.81
**Age (year)**	11.7 ± 8.0	11.3 ± 5.0	0.36	21.7 ± 10.1	20.3 ± 9.6	0.56
**GLS**^**a**^	−16.8 ± 3.6%	−21.3 ± 4.0%	*0.0002*	−16.2 ± 5.4%	−21.6 ± 3.7%	*< 0.0001*
**GCS**^**a**^	−22.5 ± 5.2%	−21.1 ± 3.3%	0.28	−20.9 ± 6.8%	−21.9 ± 3.5%	0.42
**GRS**^**a**^	21.8 ± 11.0%	26.9 ± 7.7%	0.07	22.5 ± 10.0%	27.3 ± 8.3%	*0.03*
	*early photon*	*early proton*	*p-value*	*late photon*	*late proton*	*p-value*
**GLS**^**b**^	−16.4 ± 2.3%	−17.6 ± 5.1%	0.50	−15.9 ± 5.3%	−16.8 ± 6.3%	0.78
**GCS**^**b**^	−22.0 ± 5.9%	−23.4 ± 3.6%	0.64	−20.6 ± 7.4%	−21.6 ± 4.6%%	0.77
**GRS**^**b**^	22.5 ± 11.5%	20.4 ± 10.9%	0.74	22.1 ± 8.8%	24.4 ± 16.0%	0.65
	*early < 30 Gy*	*early* ≥ *30 Gy*	*p-value*	*late < 30 Gy*	*late* ≥ *30 Gy*	*p-value*
**GLS**^**c**^	−17.1 ± 3.9%	−17.1 ± 3.6%	0.50	−15.8 ± 4.5%	−14.9 ± 4.2%	0.72
**GCS**^**c**^	−24.0 ± 5.2%	−20.6 ± 5.9%	0.64	−24.0 ± 8.4%	−20.0 ± 7.1%	0.31
**GRS**^**c**^	26.8 ± 10.1%	15.7 ± 9.1%	0.74	21.3 ± 12.9%	21.2 ± 8.3%	0.99

^a^N available for analysis: early = 18 GLS, 15 GCS, and 16 GRS; late = 26 GLS, 29 for GCS and GRS

^b^N available for analysis: early photon = 11 GLS and GRS, 10 GCS; early proton = 7 GLS, 5 GCS and GRS; late photon = 23 GLS, 24 GCS and GRS; late proton = 4 GLS, 5 GCS and GRS

^c^N available for analysis: early < 30 = 8 for all; early > 30 = 8 GLS, 5 GCS, and 6 GRS; late < 30 = 4 for all; late ≥ 30 = 20 GLS, 22 GCS and GRS

## Discussion

Craniospinal irradiation is a common therapeutic option for patients with CNS malignancies. Traditionally, CSI has been associated with improved survival rate in these patients, however it is believed that long-term effects include remodeling of the myocardial extracellular matrix yielding fibrosis of the surrounding cardiac tissue [14, 15]. Recent data from the Childhood Cancer Survivor Study showed a significant reduction in coronary artery disease and a non-significant reduction in cardiomyopathy for survivors of pediatric cancer, largely attributed to historical reductions in cardiac exposure to radiation [16]. In addition, efforts have been made to decrease cumulative doses of cardiotoxic chemotherapy agents such as anthracyclines. Despite attempts to reduce therapy while maintaining disease control, survivors of pediatric cancer may show evidence for ventricular dysfunction or subclinical cardiotoxicity [17].

The present study sought to examine the degree of left ventricular systolic dysfunction as measured by both conventional and speckle tracking echocardiography in patients undergoing CSI. When compared to an age-matched control group, patients at both early (< 12 months after therapy completion, mean 0.22 years) and late (≥ 12 months after therapy completion, mean 13.1 years) time points demonstrated depressed GLS in the presence of normal left ventricular EF and SF, which is evidence of subclinical myocardial dysfunction. This is in line with previous studies showing GLS as a marker of dysfunction in patients receiving chemotherapy with otherwise normal EF. For other strain parameters, GCS was no different at any time point and GRS only showed a decrease at the late time point. Analyses were also performed to determine whether dose or type of radiation were important in these changes in strain. There were no significant differences based on these parameters, however the numbers available for analysis in the sub-groups were small making definitive conclusions suspect.

One potential confounding factor in the present analysis is the concomitant use of chemotherapy with CSI, particularly cyclophosphamide which is known to be cardiotoxic. Although there are certain young patients with specific tumors that may be treated with high dose chemotherapy and stem cell transplant rescue, radiation is the standard of care in this population and in our sample the vast majority also received chemotherapy. Analysis of patients who did not receive radiation as part of their treatment may help resolve how much of a role chemotherapy played in this cohort, however in this retrospective study numbers were expected to be too small for meaningful analysis.

The diagnosis and management of cardiovascular injury in pediatric patients undergoing CSI may be delayed due to the misconception that this particular cancer-directed therapy results in only long-term cardiovascular side effects due to the absence of studies documenting the clinical and advanced imaging manifestations from CSI. Indeed, current guidelines in pediatrics for the surveillance of cardiovascular disease in patients undergoing CSI call for screening many years after completion of therapy. Strain analysis during the past decade has facilitated significant advances in noninvasive myocardial mechanics and cardiac function assessment [18]. Myocardial strain can assess myocardial deformation longitudinally, circumferentially, and radially, as well as in the form of twist or torsion. This would allow generation of data that increase the cardiac phenotype in patients while assessing early subclinical dysfunction in those receiving treatment, including CSI [18–20]. Whether this will lead to improved outcomes, particularly in patients undergoing CSI, needs to be determined and is worthy of further investigation.

The current study has several limitations. First, the patients included were treated over a 2-decade period. Therapeutic approach in that time has evolved, and an overall decreased scatter in radiation doses has limited the incidence of cardiotoxicity, and this could decrease the signal for cardiotoxicity in patients treated in the recent era [16]. No significant difference was detected between our subset of patients who received proton therapy vs. photon at either time point, but numbers were too small for meaningful conclusions. Second, while myocardial strain analysis has been available for better than a decade, it has yet to be regularly employed in pediatric surveillance, and for studies from the first part of the period of study in this cohort there may have been incomplete imaging planes captured to allow for retroactive strain analysis. This contributed to having fewer patients available for strain analysis than for standard echocardiographic assessment. While using a vendor-independent software theoretically limits differences for strain values between different ultrasound image vendors, differences in agreement have been reported [21]. Because this was a retrospective study covering several years, there was no ability to control for the ultrasound image vendors used without significantly limiting the number of studies available for analysis; future studies will take this into consideration. Finally, because echocardiography has not been a regular part of management of this patient group, there were limited cases in which an echocardiogram was performed in the early period after treatment, and even fewer in which a baseline echocardiogram was performed. Simply put, we could not determine if there was a baseline level of cardiac disease in this patient population prior to therapy, nor did we have an appropriate number of patients with studies both early and late after therapy to determine if there was worsening or improvement with time. It is tempting to assume there is no significant disease in this population prior to therapy, however in patients with leukemia there is a baseline increase in troponin and natriuretic peptide that is improved shortly after cancer therapy is initiated, suggesting an underlying state of cardiac stress related to the illness [22]. Additionally, measures of diastolic function were not included until the most recent few years, and this is known to be an important component to ventricular dysfunction caused by radiation therapy. It may be the case that diagnosis of CNS tumor alone can affect cardiac function, and future studies must include baseline studies prior to starting therapy.

## Conclusion

Subclinical left ventricular systolic dysfunction demonstrated by GLS is present both early and late after CSI. There was not a clear association between dose and/or type of radiation therapy, however numbers in these sub-groups were small and may not have been enough to find a difference if present. These results argue for future prospective studies to determine baseline cardiovascular disease burden and need for early initiation of longitudinal follow-up in CNS tumor patients post CSI. Coupled with cardiac biomarkers, this may allow a more complete phenotype of injury related to treatment of CNS tumors in pediatric patients.

## Data Availability

The datasets during and/or analyzed during the current study available from the corresponding author on reasonable request.

## Abbreviations

CSI: Craniospinal irradiation
CNS: Central nervous system
EF: Ejection fraction
GCS: Global circumferential strain
GLS: Global longitudinal strain
GRS: Global radial strain
MRI: Magnetic resonance imaging
SF: Shortening fraction
